# Social Development of Adults with Autism Spectrum Disorder During Dog-Assisted Therapy: A Detailed Observational Analysis

**DOI:** 10.3390/ijerph17165922

**Published:** 2020-08-14

**Authors:** Carolien Wijker, Steffie van der Steen, Annelies Spek, Ruslan Leontjevas, Marie-Jose Enders-Slegers

**Affiliations:** 1 GGZ Oost Brabant, Berlicumseweg 8, 5248 NT Rosmalen, The Netherlands; 2 Faculty of Psychology and Educational Sciences, Open University, Valkenburgerweg 177, 6419 AT Heerlen, The Netherlands; roeslan.leontjevas@ou.nl (R.L.); marie-jose.enders@ou.nl (M.-J.E.-S.); 3 Department of Special Needs Education and Youth Care, Faculty Behavioral and Social Sciences, University of Groningen, Grote Kruisstraat 2, 9712 TS Groningen, The Netherlands; s.van.der.steen@rug.nl; 4 Autism Center of Expertise, Goyergracht Zuid 39, 3755 MZ Eemnes, The Netherlands; anneliesspek@hotmail.com

**Keywords:** autism, adults, dog-assisted therapy, social development, working mechanism

## Abstract

Social communication and self-esteem are often affected in adults with autism spectrum disorder. Implementation and evaluation of interventions targeting social skills are challenged due to specific characteristics of autism. Intensive, valid evaluation of social skills programs is needed. In this explorative multiple case study, we examined effects and working mechanisms of dog-assisted therapy on social communication and self-esteem, by analyzing detailed observations with Monte Carlo permutation tests (testing against 10,000 random samples) and using self- and other-reports in N=6 high-functioning adults with ASD. Results showed significant positive effects on secure body posture. There was an indication of improved self-esteem and more spontaneous touching of the dog, while no convincing increase was found for verbal initiatives. Cross-correlation analyses revealed that touching the therapy dog may be an important determinant to elicit social development in Animal Assisted Therapy (AAT). Considering preliminary results, we recommend exploring underlying mechanisms more thoroughly with real-time observations, accounting for possible gender-effects.

## 1. Introduction

Problems in social functioning and negative social experiences are common in adults with autism spectrum disorder (ASD) [1]. This poses high risk for social isolation, poor self-esteem, stress and depression [2,3,4]. To reduce these problems, several social skills interventions have been developed, mostly for children and young adults with ASD (e.g., PEERS) [5]. It is important to realize that implementing learned social skills into daily life can prove challenging, due to multiple characteristics of ASD [6,7]. For example, the elevated stress often experienced in social contact [8] can cause information overload [9], jeopardizing recall and reproduction of the learned skills. Impairments in imagination and generalization [10,11] also limit intervention effects [12]. What happens during therapy may be considerably different from what happens in daily life. For example, role-play exercises may be perfectly executed during a therapy session, when dialogues for specific social interactions can be carefully scripted and memorized in a step-by-step fashion. However, daily life situations do not usually afford the same opportunity to prepare for spontaneous conversations. Therefore, the execution and outcomes in ordinary life may be quite different from the more controlled setting of therapy. In addition to this limitation, role-play exercises have been evaluated as stressful [13], and stress might have a negative effect on the feasibility of an intervention. Non-stressful learning contexts are required for effective training of social skills in ASD.

Animal Assisted Therapy (AAT) is an intervention that has been evaluated as both feasible and relevant for adults with ASD, and which has also been shown to be effective on stress reduction and improvement of social communication [13,14]. AAT is a structured, goal-oriented intervention in which a trained animal plays an integral role [15]. Although positive intervention effects for ASD (mostly in children) have been described in AAT literature, several authors have warned about specific methodological limitations [16,17]. For example, assessment of social functioning in the ASD population can prove challenging, due to the lack of sensitive outcome measures. Questionnaires are prone to positive illusory, social desirability bias, and rater biases, especially in the ASD population [18,19]. Therefore, evaluation of social skills interventions is difficult [20]. Behavioral observations overcome some of these problems. Since daily life interactions involve dynamic patterns between a person and his or her environment [21], observations of, for example, number of words spoken [22,23] lack the sensitivity needed to capture real-life social functioning, especially in the high-functioning adult ASD population. Real-time observations of dynamic interactions are necessary to draw valid conclusions about an intervention, and such observations may help researchers explore its working mechanisms [24].

To date, the working mechanisms of AAT remain largely unknown [25]. Understanding these mechanisms is essential for refining social skills interventions and choosing the most suitable exercises to optimize effects in daily life. It has been hypothesized that stress reduction plays a key role in AAT [26], and research has shown that hormones associated with stress in humans decrease after a person has petted a dog [27,28]. Furthermore, it has been argued that dogs can catalyze social communication in humans, as their presence facilitates joint attention and provides a concrete subject of conversation [29]. It has also been proposed that positive social interactions and specific exercises, aiming to increase awareness about one’s body language, such as facial expression, posture or gestures in relation to others, reinforce positive self-perception and confident thoughts [30,31,32]. These embodied cognitions positively affect self-esteem [33,34].

Intensively studying data from a smaller sample size has the advantage over large RCT’s of obtaining detailed information and understanding of the working mechanisms of an intervention. In this study we aimed to understand the effects and mechanisms of a dog-assisted therapy in adults with ASD, through detailed real-time observations targeting social communication and self-esteem. Our study is, to our knowledge, the first to explore effects and mechanisms of AAT using detailed real-time observations in a high-functioning adult ASD subsample.

The following research questions were answered:

(1) Are improvements in social communication and self-esteem observed in the AAT intervention condition and are the observed variables evaluated as valid?
(2) Are the real-time observed behaviors related?
(3) Are dynamic patterns (one behavior proceeding another behavior) observed in the behaviors of the participants during AAT sessions?

## 2. Materials and Methods

### 2.1. Design

For this particular study, we approached participants who were involved in a larger Randomized Controlled Trial on AAT for adults with ASD [35]. This study was conducted between January 2015 and July 2017 at the mental health care organization GGZ Oost Brabant, The Netherlands. The study was approved by the medical ethics committee CMO and was registered in the Dutch Trial Registry (NTR5938).

After recruiting, a baseline assessment was conducted (including self-reports and physiological measures of stress; more details on this assessment are described in our study protocol [34]), and then participants were randomly assigned to a control or AAT group. The AAT group received 10 weekly sessions of AAT with a professional therapist and therapy dog (see below for a description). Post-treatment assessment (similar measurements as the baseline assessment) was conducted 10 weeks after baseline. Within the AAT group, we asked a small number of participants (N = 6) if we could film the therapy sessions to observe their behavior. The current study focuses on these six participants.

### 2.2. Participants

Participants were recruited from the mental health care organization and the Dutch Society for Autism (Nederlandse Vereniging voor Autisme, NVA). Information flyers were placed in the waiting room, and verbal information was provided by therapists. When prospective participants were interested in the study, they were screened for eligibility. Prospective participants were eligible for the current study when they: (1) were diagnosed with autism spectrum disorder, which was evaluated by a combination of the Autistic Disorder Interview-Revised [36], a semi-structured interview to assess the DSM-5 criteria [37], and clinical observations, (2) were between 18 and 60 years of age, (3) had an IQ score above 80 on the Wechsler Adult Intelligence Scale III or IV [38,39], and (4) scored higher than 132 on the Symptom Checklist-90-Revised [40] and higher than 19 on the Perceived Stress Scale [41].

Prospective participants were excluded when they: (1) met the criteria for a current psychosis or suicide risk, as indicated by their psychologist or psychiatrist, (2) were institutionalized, (3) were allergic to dogs, afraid of dogs, or had an aversion to dogs, and (4) received other treatments during the study period.

All participants in the current study signed an informed consent and were aware that they could withdraw from the study at any moment without providing an explanation. Participant selection for the current study was non-random. Selection was based on schedule compatibility between the participant and the research assistant who was video-recording the sessions.

Baseline characteristics of the participants are shown in Table 1. The sample consisted of two females, each of whom lived with a partner and children, and four males, one of whom lived with his father, and three of whom lived alone. One female participant had a dog at home at baseline measurement. One participant (participant 6) showed high total and verbal IQ scores, while the other five participants had average IQ scores (varying from 91 to 107).

### 2.3. Animal Assisted Therapy (AAT) Program

Participants received 10 weekly one-on-one sessions of dog-assisted therapy guided by a certified healthcare professional with a college or university degree and a minimum of five years working experience with adults with ASD. To create a safe working environment for both humans and animals during the therapy sessions, the therapists in this study also completed courses on dog welfare and dog training. The therapy dogs working in this program (two poodles and two Labrador crossbreeds) were selected from and trained by the Dutch service dog foundation Hulphond Nederland. Hulphond Nederland also formulated guidelines for this specific therapy program to protect and monitor animal welfare (e.g., a maximum of two non-consecutive working hours per day, and a maximum of two days per week depending on the breed, age, and fitness of the dog).

The aim of the AAT was to reduce stress and improve social and communication skills by increasing self-insight. During the sessions, the therapist, participant and therapy dog worked in a triad. An important role of the therapist was to verbalize and subtitle behaviors and interactions in the triad. The therapist provided insight and knowledge into thoughts, feelings and behaviors and facilitated the interactions in the triad. For example, when the therapist noted that the dog was distracted during a walking exercise and the participant did not seem to be aware of this, the therapist would ask questions to raise the participant’s awareness (i.e., ‘What do you think the dog is thinking right now?’, ‘Where is the dog looking at?’, ‘How do you feel when the dog is not paying attention to you’? or ‘What can you do to improve the interaction’?). The dog was present during the sessions to assist during the exercises on one hand (for example, to execute the walking exercise together with the participants) and for provision of social support and comfort on the other hand (the possibility to pet the dog, playing games with the dog).

All therapy sessions had a duration of 60 min each. Sessions were structured with 5 to 10 min of reflection and repetition of exercises from the previous week, followed by introduction, execution, and reflection of new exercises. Examples of exercises are: observation and interpretation of the behaviors of the dog, basic instructions such as ‘come’, ‘sit’ and ‘paw’ (verbal and non-verbal), guiding the dog without a leash through an obstacle course (with and without verbal guidance), and outdoor walking with the dog (on a leash). During the last 10 to 15 min of each session, participants were provided time for physical interaction with the therapy dog in the form of petting or grooming the dog. During this interaction time, the therapist withdrew in order to optimize contact between the participant and the dog. In the last few minutes of the session, the therapist and the participant briefly reflected on the most important insights of the therapy. Tools were provided for implementation of the learned skills into daily life, such as ways to incorporate the learned skills to manage stressors. More detailed information of the AAT program, animal welfare, ethics and guidelines is written elsewhere [13,35]. No animals were harmed during the study.

### 2.4. Instruments

#### 2.4.1. Behavior Observations

In this study we aimed to understand the effects and mechanisms of a dog-assisted therapy in adults with ASD, through detailed real-time observations targeting social communication and self-esteem. Social communication was measured by observation of spontaneous touch and verbal initiative [37,42]; self-esteem was measured by observation and evaluation participant’s posture [30,32]. Spontaneous touch was coded when the participant touched or stroked the dog. Touch was not coded when the therapist suggested that the participant could stroke the dog, when it was part of the exercise (practicing the command “give paw”), or when the dog was rewarded with a treat.

Verbal initiative was coded when spontaneous verbal initiations toward either the therapist or the dog were observed. Examples of such behavior include spontaneously starting a conversation, asking a question, changing the subject or adding new elements to an existing conversation. Responses to the therapist’s questions or to the dog’s actions were not coded as verbal initiative, since we only scored spontaneous verbal initiatives.

Lastly, posture was coded by assessing the average level of confidence of the participant every five minutes. The scores ranged from 1 to 5. Score 1 (highly insecure) was coded when the participant did not seem comfortable, such as when the participant looked away or at the ground, had a rigid posture, showed no variations in movement, or was fidgeting. Score 2 (insecure) was coded when the participant seemed hesitant or not fully committed, with only a few variations in movement and a slightly rigid or wobbly posture. Score 3 (neutral) was coded when the participant performed the exercises and seemed committed, with brief moments of hesitation or insecurity. Shoulders were straight most of the time and variations in movement were observed. Score 4 (secure) was coded when the participant had an upright posture, and actively participated during the exercises, with a higher level of energy compared to that of score 3. Only very brief moments of insecurity could be observed. The participant varied in movement and also in connecting to the therapist or dog. Score 5 was coded when the participant had an upright posture and variations in movement and gaze direction, and when he/she seemed to fully enjoy the exercises. Moments of insecure posture were absent, and the interaction with therapist and dog seemed natural.

#### 2.4.2. Questionnaires

Three subscales (social awareness, social communication, and social motivation) of the Dutch version of the Social Responsiveness Scale for Adults (SRS-A, self-report version) [43] were used to obtain self- and other-reported information for social communication. Items were rated on a four-point scale ranging from 1 (not true) to 4 (almost always true). Higher total scores on the subscales corresponded with more impairments in social communication. The Dutch version has good internal consistency and test-retest reliability, and a sufficient intraclass correlation coefficient for the self-report and informant report [43].

Self-reported self-esteem was measured using the Rosenberg Self-Esteem Scale (RSES) [44]. The questionnaire contains ten items, each rated on a four-point Likert scale ranging from 1 (very untrue) to 4 (very true). A higher total score corresponds with higher rating of self-reported self-esteem. This instrument showed high internal consistency and test-retest reliability [45].

### 2.5. Procedure

At baseline (T0) and post-intervention (T1), participants were asked to fill out both questionnaires (RSES and SRS-A self-report) at the mental health care organization. A research assistant checked the questionnaires for missing items and asked participants to complete any missing information before leaving the assessment room. A spouse, close family member or friend of the participant was also asked to fill out the SRS-A other-report to evaluate the participant’s social communication.

Each participant’s first therapy session (S1) and last therapy session (S10) were recorded on video by a research assistant. Two cameras were placed in the corners of the therapy room to capture the entire space from different angles. Recordings began the moment the participant entered the room and continued through the end of the session. During an outdoor exercise in the final session (S10), a research assistant carried the camera at an appropriate distance in front of the participant to follow his or her movements.

The three observed variables, verbal initiative, spontaneous touching and posture were coded with a time stamp using the program Mediacoder [46]. Variables were coded in real time, that is, continuously during the full interaction. Four master’s students were involved in coding and used a standardized coding protocol with guidelines and examples to ensure reliability. The students first underwent a training protocol in which the coding rules were explained, and coded a video of 20 min for each variable separately. The codes were then compared and percentages of agreement were calculated. When calculating percentages of agreement, both the given codes, as well as the timing were taken into account. Differences in timing of less than two seconds were considered acceptable. When a cut-off percentage of 80% agreement was not reached in the first round of training, the rules were further explained and specific examples were shown. Then another video was coded to determine inter-rater agreement. The final percentages of agreement were 80% for spontaneous touch and verbal initiative, and 91% for posture.

### 2.6. Analyses

To answer the first research question: “Are improvements in social communication and self-esteem observed in the AAT intervention condition and are the observed variables evaluated as valid?” we calculated average scores of the participants (N=6) on observed behavior and outcomes of self- and other-reports, targeting social communication and self-esteem.

#### 2.6.1. Observations

First, we controlled for the length of the sessions, as this varied among participants. The number of observed behaviors (for verbal initiative and spontaneous touch as this was expressed in frequencies) was divided by the total time (in minutes) of the session and multiplied by 100 to calculate percentages.

We used macros in Microsoft Excel 2016, version 1.5 (Microsoft, Redmont, WA., USA), to conduct Monte Carlo permutation tests [47] to compare the average percentages of the observed variables between the first session (S1) and the final session (S10). Monte Carlo permutation tests are especially suited for small sample sizes. We used the actual sample distribution as input, to determine the probability that a difference (S1–S10) was caused by chance. For the procedure, we took 10,000 random samples from the original data and aimed to determine how often the observed difference occurred in these. A *p*-value was calculated by dividing this occurrence by the number of random samples (10,000).

#### 2.6.2. Questionnaires

Using Monte Carlo permutation tests, we compared average scores of the SRS-A (self- and other-report) and RSES between T0 and T1. For one participant (participant number 5) the informant version of the SRS-A was not completed and returned to the principal researcher. These data were regarded as missing and were not imputed. Therefore, the average SRS-A other-report was calculated with N = 5 participant’s scores.

#### 2.6.3. Relation Between Observed and Self- and Other-Reported Skills

We visually inspected the relationship between the observations and corresponding outcomes on questionnaires to validate our observations. Mean scores of social interactions (SRS-A, self- and other-reports) were graphically plotted as a function of mean scores on verbal initiative and spontaneous touch respectively. The T0 baseline scores of the questionnaires were used as approximation of the scores of the observed variables from the first session (S1), and the T1 post-intervention scores were used as approximation of the scores of the observed variables from the final (tenth) session (S10). We followed the same procedure for mean RSES scores and observed posture. The plots were visually checked regarding similarity in direction of change of corresponding variables and verified by the Monte Carlo permutation test scores. A global description is provided in the Results section.

To answer the second research question: *“Are the real-time observed behaviors related?”* we created data points by making a time series of one second for each variable (verbal initiative, spontaneous touch and posture) and graphed these points in time order. Data were smoothed using a local regression (LOESS) function with a 10% window to create a waveform graphical representation of the observed behaviors as a function of time. This nonparametric technique can be used to represent relationships between variables [48]. To visually inspect the relations and dynamic patterns of the behaviors during the therapy sessions, we created graphical representations for the observed variables in both sessions of the participants, with the time in seconds on the X-axis and the smoothed data values on the Y-axis.

We used cross-correlation analysis [49] to measure the similarity between two observed behaviors as a function of time by calculating the relationship between the two waveforms plotted for each participant. Pearson correlation coefficient (*r*) was calculated for each combination of the three behaviors; *r* of <0.3 (negligible), between 0.3 and 0.5 (low), between 0.5 and 0.7 (moderate), between 0.7 and 0.9 (high), and between 0.9 and 1 (very high) [50]. Due to the absence of spontaneous touching of the therapy dog during the first session (S1) for participant 4, the overall correlations for the combinations including spontaneous touch could not be calculated for this participant.

To determine the relationship between each combination of two variables as a function of time, we performed a windowed correlation analysis. Time windows of 30 s were created for each combination of the three variables. We then calculated the percentage of positive windowed correlations for each combination of two variables. A positive windowed correlation was appointed when two variables were both present or absent in the same window. Only positive windowed correlations >50% were considered, as this means that in more than half of a session, two behaviors were both present or absent at the same time.

To answer the third research question: “Are dynamic patterns (one behavior proceeding another behavior) observed in the behaviors of the participants during AAT sessions?” we performed a cross-correlation forecasting analysis [49]. A case description and graphical representation of a dynamic behavioral pattern will be presented in the Results section for visual illustration of behavioral patterns within a therapy session.

To study the dynamic relation between the variables, we calculated the correlation of two variables by shifting the 30-s time window of two variables relative to each other, with a range between −240 and +240 s. A higher correlation after a time shift was regarded as an indication for a possible dynamic behavioral pattern (one behavior proceeding the other behavior). The highest correlation between the two variables with a range between −240 and +240 s was noted.

For interpretation of the data, we considered only correlations within time delays between −120 and +120 s, as two consecutive behaviors observed in a time frame outside 120 s are more likely to be a random occurrence than a meaningful behavioral pattern.

## 3. Results

### 3.1. Changes in Social Communication and Self-Esteem

Results on question 1“Are improvements in social communication and self-esteem observed in the AAT intervention condition and are the observed variables evaluated as valid?” are presented in Table 2.

#### 3.1.1. Observations

Monte Carlo permutation tests showed higher scores for secure posture during the last session (S10) compared to the first session (S1) (S1, 2.44; S10, 3.44; *p* = 0.007). Verbal initiatives toward the therapist and therapy dog remained relatively stable on the group level between the first session (S1) and the last session (S10) (S1, 77%; S10, 74%; *p* = 0.543). The amount of spontaneous touching of the therapy dog, although not reaching significance levels (*p* = 0.140), increased from 16% in the first session (S1) to 24% in the last therapy session (S10). High intra- and inter-individual variation was found for this variable with percentages varying between 0 and 44. It was notable that the amount of spontaneous touching of the therapy dog was highest in females during the first session (S1) and decreased for both females in the final session (S10). Opposite results were found in the male participants, all of whom showed increased spontaneous touching of the therapy dog in the final session (S10) compared to the first session (S1).

#### 3.1.2. Questionnaires

Although Monte Carlo analysis did not reach significance level, yet improvements were observed in self-esteem (RSES, *p* = 0.116). Social interaction and communication did not show significant changes on group level for these six participants after 10 AAT sessions (SRS-A self-reports, *p* = 0.582; SRS-A other-reports, *p* = 0.375).

#### 3.1.3. Relation Between Observed and Self- and Other-Reported Skills

Observed verbal initiative and posture seemed to be in accordance with the outcomes on questionnaires targeting corresponding constructs. As posture improved significantly, correspondingly, self-reported self-esteem, although not reaching significance levels, improved. Spontaneous touch, although not reaching significance levels, increased, while no changes were observed in self- and other-reported social communication. No significant changes were found in verbal initiative towards the therapist and therapy dog between the first session (S1) and the last session (S10), which was in line with self- and other-reported social communication.

### 3.2. Relation Between Observed Variables

Results for question 2 *“Are the real-time observed behaviors related?”* are presented in Table 3, Table 4 and Table 5.

#### 3.2.1. Overall Correlations

The highest correlations were found for combinations that included spontaneous touch. All moderate/high correlations were found in the final therapy session (S10). Our analysis showed that the correlations between “verbal initiative and posture” were negligible to low (-0.419< *r* >0.439) (Table 3). The combination “verbal initiative and spontaneous touch” showed no association for two participants (participant 3 and 4). For three participants, the correlation increased from low/moderate to moderate/high during the last therapy session (S10). Two moderate correlations (*r* = 0.524 and *r* = 0.563) were found in two participants (participants 5 and 6, respectively) and one high overall correlation (*r* = 0.774) was found in one participant (participant 1) (Table 4).

Regarding the combination “posture and spontaneous touch”, for two participants (participant 2 and 3) the correlation was negligible in both sessions (S1 and S10). For one participant, the correlation decreased from low (*r* = 0.458) in the first therapy session (S1) to negligible (*r* = 0.235) in the last therapy session (S10). For two out of six participants, the correlation increased from negligible/low to moderate correlations (*r* = 0.657 and *r* = 0.560; participants 5 and 6, respectively) during session 10 (S10) (Table 5).

#### 3.2.2. Windowed Correlations

Windowed correlation analysis showed a percentage of >50% for all three combinations of the observed variables in five out of twelve therapy sessions (Table 3, Table 4 and Table 5). Overall, the highest positive windowed correlations were found for verbal initiative and spontaneous touch, indicating that both behaviors were similarly absent or present during the sessions. For the combination “verbal initiative and posture”, windowed correlation analysis showed positive correlations of >50% in four out of six participants (participants 1, 2, 4, and 5) during the first session (S1) (Table 3). During the last therapy session (S10), positive windowed correlations of >50% were found in five of the six participants (participants 1, 2, 3, 4, and 6). For the combination “spontaneous touch and verbal initiative”, positive windowed correlations of >50% were found in four out of six participants (participants 1, 2, 3, and 6) during the first therapy session (S1) and the last therapy session (S10) (Table 4). Furthermore, positive windowed correlations of >50% were found for the combination “spontaneous touch and posture” in four out of the six participants (participants 1, 2, 3, and 5) during the first therapy session (S1) and in four participants during the last therapy session (S10) (participants 1, 3, 5, and 6) (Table 5).

### 3.3. Dynamic Patterns

**Figure 1:**
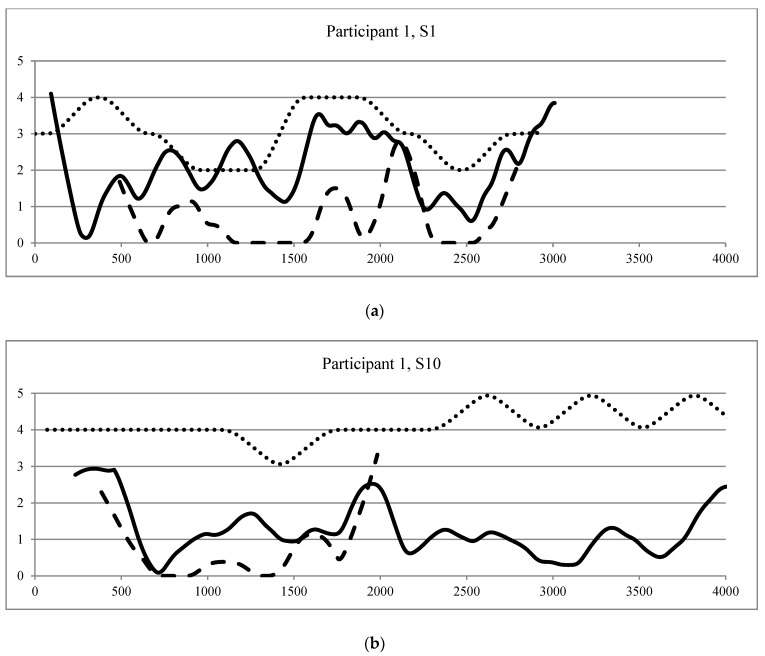
(**a**) Graphic representation of the first therapy session (Notes: Data points of participant 1 during the first therapy session (S1), smoothed with LOESS as a function of time (in seconds). Legend: —Verbal initiative; —Spontaneous touch; …… Posture); (**b**) Graphic representation of the last therapy session (Notes: Data points of participant 1 during the last therapy session (S10), smoothed with LOESS as a function of time (in seconds). Legend: —Verbal initiative. —Spontaneous touch……Posture).

Results of question 3 “Are dynamic patterns (one behavior proceeding another behavior) observed in the behaviors of the participants during AAT sessions?” are presented in Table 3, Table 4 and Table 5. Graphical representations of the observed behaviors as a function of time are presented for one participant in Figure 1a,b, illustrating the dynamic movement of the variables during the AAT sessions.

#### 3.3.1. Case Description of a Dynamic Behavioral Pattern

In the first therapy session (S1), a dynamic behavioral pattern was observed with changes in posture proceeding changes in verbal initiative and changes in verbal initiative proceeding changes in spontaneous touching of the dog. Figure 1a shows that during this session, long periods of simultaneous behaviors (absent or present at the same time) were observed between spontaneous touch and verbal initiative. Furthermore, the graph shows that changes in verbal initiative were followed by changes in spontaneous touch, with a little time delay: increases in verbal initiative were followed by an increase in spontaneous touching of the therapy dog and decreases in verbal initiative were followed by a decrease of spontaneous touch. A more stable pattern was observed for posture during the session, yet between approximately 1500 and 2000 s, a simultaneous pattern was observed between posture and verbal initiative. Within this time frame, the graph shows that a more secure posture was followed by an increase of verbal initiative, and a less secure posture was followed by a decrease of verbal initiative.

In the last therapy session (S10), changes in spontaneous touch occurred prior to changes in posture. Figure 1b shows that spontaneous touch only occurred during the first half of the therapy session (up until approximately 2000 s). During this period, behavioral patterns between spontaneous touch and verbal initiative and dynamic behavioral patterns were observed for spontaneous touch and posture. Verbal initiative and spontaneous touch showed simultaneous patterns, with both behaviors being absent or present at the same time. A dynamic behavioral pattern was observed for spontaneous touch and posture. Increases in spontaneous touching of the therapy dog were followed by a more secure posture and decreases in spontaneous touch were followed by a more insecure posture.

#### 3.3.2. Cross Correlations

Overall, for seven out of twelve therapy sessions, a shift in time for one variable (ranging from 12 to 113 s) increased the overall correlation between two variables (Δ*r* = 0.003 to Δ*r* = 0.314). In more than half of all sessions, dynamic behavioral patterns seemed to occur, with one behavior proceeding another behavior.

The cross-correlation analysis showed a dynamic pattern for the combination “posture and verbal initiative” in participant 1 during the first session (S1). Changes in posture occurred prior to changes in verbal initiative with a 12-s delay, resulting in a maximum correlation of *r* = 0.345 (Table 3). For the combination “spontaneous touch and verbal initiative”, we observed a dynamic pattern with changes in verbal initiative proceeding changes in spontaneous touch (Table 4). We observed this pattern in three participants: in participants 1 and 3 during the first session (S1) (with a 72- and 106-s delay, respectively), and in participant 5 during the last session (S10) (with a 103-s delay). For the combination “spontaneous touch and posture”, we observed a dynamic pattern of changes in posture proceeding changes in spontaneous touch in participants 1 and 5 during the first session (S1) (with an 87- and a 29-s delay, respectively) and in participant 6 during the last session (S10) (with a 67-s delay). We observed an opposite pattern of changes, with spontaneous touch proceeding changes in posture in participant 1 during the last session (S10) (with a 113-s delay) and in participant 2 during the first session (S1) (with a 22-s delay).

## 4. Discussion

The current study aimed to explore effects and working mechanisms of an AAT in adults with ASD, targeting social communication and self-esteem by detailed, real-time observational analyses. Data showed that AAT had a significant effect on observed posture, which was rated as more secure in the final session compared to the first session. The results were in line with self-reported self-esteem in which, although not significantly, a positive trend was shown. Research showed that a more upright posture is related to more confident thoughts [30]. In several exercises of the AAT protocol [35], this principle was used with the aim of improving body-awareness and self-esteem. For example, participants were asked to guide the therapy dog without a leash through an obstacle track. The dogs showed more desirable responses when participants walked with a greater confidence and an upright posture. Kruger and Serpell [31] argue that social cognition and perception, including self-esteem, can improve in humans as a result of immediate and unambiguous feedback from animals. Therefore, a more upright body posture might have reinforced more desirable responses (for example, increased attention to the participant) of the dog and these responses might have led to increased self-confidence and higher self-esteem. These results are important for clinical practice, as the most prevalent comorbid problems in adults with ASD—anxiety and depression—are strongly associated with low self-esteem [4]. In future research, the effects of AAT on self-confidence, self-esteem and body posture should be explored in greater detail. In clinical practice, this information is important for dog selection (e.g., the dog’s obedience and focus on handlers, etc.) and specific protocol exercises for patients with lower self-esteem.

The RCT showed significant improvements in proxy-rated social communication [14]. In this study, however, no significant changes were found for outcomes targeting social communication. Because of the large variations observed in verbal initiative, suggesting a considerable interpersonal diversity, it might have been difficult to detect changes in this small study sample. Future studies with larger sample sizes are recommended to explore effects on observed verbal initiative more excessively. Although the observed spontaneous touching of the therapy dog did not reach significance levels, most participants touched the dog more frequently during the final session (S10) compared to the first session (S1). Literature shows that touching a dog reduces hormones associated with stress and increases hormones associated with social bonding in both the animal and human [27,28]; our results might therefore indicate increased bonding, confidence or familiarity between the therapy dog and participants at the tenth therapy session. Furthermore, although the study sample was too small to draw conclusions on gender-effects, the differences in touch observed in males and females are notable. The frequency of spontaneous touch decreased in all female participants and increased in all male participants. These differences are interesting, especially when viewed in context of social camouflaging, a phenomenon whereby a person masks and compensates for his or her difficulties. Having to pretend to be “not-autistic” is exhausting and stressful and can affect one’s sense of identity [51]. Females with ASD tend to mask their social communication deficits more often compared to males [52]. Camouflaging in females is positively related to sensitivity and awareness of their social environment [53]. Possibly, the female participants were more aware of the dogs’ presence during the first session, resulting in more spontaneous touching. It is hypothesized that animals have unambiguous and “non-judgmental” qualities [54]. For this reason, it might be that females reduced “overcompensating” and became less (“over-”) responsive to their social environment during sessions, resulting in less touching. As camouflaging comes with high costs, the non-judgmental quality of the dog, can have resulted in diminished camouflage strategies. In contrast, high camouflaging in males has been found to be positively related to depression [51]. If the non-judgmental quality of the dog facilitated higher acceptance of ASD, there is a possibility that AAT reduces depressive symptoms in males. As one male participant reported:

“*I felt relaxed and cheerful after every session. Cuddling the dog at the end of each session miraculously changed my mood*.”

To refine AAT and optimize its effectiveness, understanding of the study’s effects and working mechanisms is important. Therefore, it would be interesting for future research to intensively examine the underlying mechanisms of AAT, accounting for possible gender effects and the role of camouflaging, as a different mechanism might be evolving in males and females.

Regarding mechanisms to explain the effects found in AAT, it has been proposed that touch plays a key role in promoting prosocial behavior [26]. In our study, of all variables observed, the combinations that included spontaneous touch showed the highest positive correlations and all were found in the final therapy session (S10). A behavior pattern of simultaneous touch and verbal initiative was found in three out of six participants, and a behavior pattern of simultaneous touch and posture was found in two out of six participants. Although these relations were not observed in all participants, touch seemed to be associated with social communication and self-esteem. This is supported by literature [42,55]. Literature shows that after touching a dog, hormones associated with relaxation, prosocial behavior and emotional bonding, are released [28]. The increased simultaneous occurrence of touch and verbal initiative, as well as increased co-occurrence of touch and a confident posture in the last therapy session, may therefore indicate the forming of a social bond between the participant and their therapy dog over the course of therapy. Furthermore, gentle, affective touch (on hairy parts of the human skin such as the forearm) triggers social domains in the brain [56]. A core symptom in ASD is hyper- or hyporeactive response to stimuli [37]. Research on touch revealed that people with ASD showed less response in the areas of the brain associated with social-emotional development after being slowly touched with a soft brush [57]. However, responses seemed to be dependent on which body part was being touched (palm of the hand versus forearm). The social regions of the brain in people with ASD were found to be more responsive to touch on palm of the hand than were social regions of the brain in neurotypical individuals [58]. Very much in line with this, as interhuman touch is often associated with stress and social withdrawal in ASD [59], touching the therapy dog (with the palm of the hand) was evaluated as relaxing and joyful [13]. This makes AAT a potentially valuable intervention for the development of social skills, especially for people with ASD, as it provides participants the opportunity to pet a therapy dog. 

With regard to behavioral patterns, we observed large intra- and interpersonal variations. Verbal initiative and body posture were not associated, which might be explained by the poor integration of verbal (initiative) and non-verbal communication (posture), one of the core deficits in ASD [37].

Given the explorative nature of this study, conclusions should be drawn with caution. While exploring behavioral patterns we found large inter- and intrapersonal variations, suggesting different therapy processes within each participant. Theories on the working mechanisms of AAT often assume that touch is the eliciting factor [26,60,61]. However, our study results showed that touch is more commonly observed after a participant has taken verbal initiative or changed his or her posture. It might be that in people with ASD, different mechanisms are at work than previously thought. For example, it may be that improvements obtained in social communication and self-esteem reduce hyper-sensitivity, resulting in increased touching. However, to examine these mechanisms more thoroughly, larger studies, including hypothesis-testing methods, are needed.

Although the nature of the study was explorative, there are some reasonable limitations in the methodology of the study that need to be kept in mind when interpreting study results. The small sample size, non-random selection of participants, and lack of a control group hamper generalizability of the study findings. Furthermore, the large inter-personal variations observed in some variables, can have jeopardized the detection of possible effects. However, we accounted for the small sample size in our analyses, yet covariates were not imputed into analyses due to the small sample size. Therefore, we were not able to draw conclusions on, for example, gender effects. Another limitation of this study is the lack of ethnic and intellectual diversity in the sample, which also limits generalization of the study results.

This is the first study to intensively explore the effects and mechanisms of AAT using detailed real-time observations in an ASD subsample. RCT’s with large sample sizes are often described as the “gold standard.” Nevertheless, intensively studying data from a smaller sample size offers a valuable opportunity to formulate hypotheses for future research and put forward guidelines for clinical practice, as provided in this paper.

## 5. Conclusions

The current study shows AAT to be a promising intervention for increasing confidence and self-esteem in adults with ASD. Due to the exploratory character of the study and the small study sample, and non-random selection of participants, results should be interpreted with caution. Results on the effects of AAT revealed a more upright and confident body posture in the last therapy session, compared to the first session. Although AAT did not seem to affect the frequency of verbal initiative and spontaneous touching of the therapy dog, we found that spontaneous touch and verbal initiative or spontaneous touch and a more secure body posture seemed to co-occur. Regarding the working mechanisms of AAT, these results seem to indicate the beneficial effects of touching the therapy dog. For this reason, exercises integrating touch with verbal initiative and body posture are recommended. Also, the diversity in behavioral patterns among participants indicates the importance of customizing the intervention for individual participants. Therapists should be aware of gender-related expressions of social communication in adults with ASD in relation to the problems and therapy goals of participants.

## Figures and Tables

**Table 1:** Baseline characteristics.

Participant	Gender	Age	Total IQ	Verbal IQ	Dog Owner	Living Situation
1	Female	36	100	98	Yes	With partner and children
2	Male	28	105	94	No	Alone
3	Male	44	103	101	No	With father
4	Male	54	97	91	No	Alone
5	Male	23	90	107	No	Alone
6	Female	42	132	127	No	With partner and children

**Table 2:** Baseline and post-treatment scores.

Participant	Timepoint	Social Communication				Self-Esteem	
		Verbal Initiative	Touch	Self-Report	Other-Report	Posture	Self-Report
1	0	119	26	74	81	3.09	24
	1	82	13	59	74	4.20	33
2	0	67	04	25	53	2.43	29
	1	73	30	29	51	3.30	34
3	0	56	14	100	94	1.92	22
	1	53	25	92	88	2.79	29
4	0	67	0	11	54	2.31	24
	1	60	23	31	55	3.62	19
5	0	18	12	60	80.12	1.80	28
	1	24	17	78	-	3.22	29
6	0	135	40	77	76	3.11	28
	1	150	33	81	75	3.50	30
**Mean group**	0	77	16	57.83	73.02	2.44	25.83
	1	74	24	61.67	68.60	3.44	29
***p*** **-value**		0.543	0.140	0.582	0.375	0.007 *	0.116

Notes: Participants’ individual and mean group scores of social communication and self-esteem per time point. * *p* < 0.05. Legend: Timepoint 0 = Baseline/ first session, Timepoint 1 = Post-treatment/ last session, Verbal initiative = number of observed verbal initiatives towards the therapist and therapy dog per minute during a session ∗ 100, Touch = number of observed spontaneous touches of the therapy dog per minute during a session ∗ 100, Self-reported Social Communication = self-reported scores on the subscales social awareness, social communication and social motivation of the Social Responsiveness Scale–Adults (SRS-A), Other-reported Social Communication = proxy-reported scores on the subscales social awareness, social communication and social motivation of the Social Responsiveness Scale–Adults (SRS-A), Posture = observed posture, Self-reported Self-esteem = self-reported scores on the Rosenberg Self-Esteem Scale (RSES).

**Table 3:** Relations between verbal initiative and posture.

Participant	Session	Overall Correlation	% Positive Windowed Correlation	Cross Correlation	Behavior Sequence	Time Shift (sec)
1	1	0.342	59.8	0.345	P → VI	12
	10	−0.136	52.1	-0.352	VI → P	210
2	1	0.014	53.7	0.023	P → VI	38
	10	−0.419	58.9	-0.481	P → VI	240
3	1	−0.025	46.4	0.556 *	P → VI	240
	10	−0.291	55.6	-0.507 *	P → VI	240
4	1	0.299	56.1	0.299	S	0
	10	0.439	50.1	0.439	S	0
5	1	0.091	50.2	0.360	P → VI	211
	10	0.332	46	0.420	VI → P	149
6	1	0.008	42.9	0.760 **	VI → P	240
	10	−0.124	52.1	-0.124	S	0

Notes: Relation between observed verbal initiative and posture during session 1 and 10. Overall correlation = Pearson correlation coefficient (*r*) of the two waveforms (data points of verbal initiative and posture (time series of one second) graphed in time order, smoothed with regression (LOESS) function), % Positive windowed correlation = percentage of positive windowed correlations between verbal initiative and posture in function of time (windows of 30 s), Cross correlation = Maximal correlation coefficient (*r*) of the cross-correlation forecasting analysis [49] with a shifted time window of 30 s relative to each other, VI = verbal initiative, P = posture. Only correlations between −120 and +120 s were considered as meaningful. S = Simultaneous behavior (when a time shift did not lead to a change in correlation-coefficient). * moderate correlation (*r* ≥ 0.5). ** high correlation (*r* ≥ 0.7).

**Table 4:** Relations between verbal initiative and spontaneous touch.

Participant	Session	Overall Correlation	% Positive Windowed Correlation	Cross Correlation	Behavior Sequence	Time Shift (sec)
1	1	0.394	52.9	0.572 *	VI → T	72
	10	0.774 **	77.3	0.774 **	S	0
2	1	0.478	70.5	0.478	S	0
	10	0.335	71.9	−0.550 *	VI → T	240
3	1	0.118	53.1	0.432	VI → T	106
	10	0.230	53.8	0.341	T → VI	240
4	1	-	-	-	-	-
	10	−0.177	48	0.410	VI → T	240
5	1	−0.237	21	−0.285	VI → T	51
	10	0.524 *	42.2	0.640 *	VI → T	103
6	1	0.394	76.6	0.394	S	0
	10	0.563 *	72.4	0.560 *	S	0

Notes: Relation between observed verbal initiative and spontaneous touch during session 1 and 10. Overall correlation = Pearson correlation coefficient (*r*) of the two waveforms (data points of verbal initiative and spontaneous touch (time series of one second) graphed in time order, smoothed with regression (LOESS) function), % Positive windowed correlation = percentage of positive windowed correlations between verbal initiative and spontaneous touch in function of time (windows of 30 s), Cross correlation = Maximal correlation coefficient (*r*) of the cross-correlation forecasting analysis [49] with a shifted time window of 30 s relative to each other, VI = verbal initiative, T = spontaneous touch. Only correlations between −120 and +120 s were considered as meaningful. S = Simultaneous behavior (when a time shift did not lead to a change in correlation-coefficient). * moderate correlation (*r* ≥ 0.5). ** high correlation (*r* ≥ 0.7).

**Table 5:** Relations between posture and spontaneous touch.

Participant	Session	Overall Correlation	% Positive Windowed Correlation	Cross Correlation	Behavior Sequence	Time Shift (sec)
1	1	0.458	65	0.494	P → T	87
	10	0.235	55.7	0.290	T → P	113
2	1	0.257	80.5	0.265	T → P	22
	10	0.048	36.2	0.540 *	T → P	240
3	1	0.119	58.2	−0.255	T → P	240
	10	0.096	65.6	−0.135	T → P	240
4	1	-	-	-	-	-
	10	−0.481	34.5	−0.560 *	T → P	58
5	1	0.380	70	0.391	P → T	29
	10	0.657 *	58.6	0.760 **	P → T	151
6	1	0.093	49.2	0.530 *	P → T	240
	10	0.560 *	63	0.620 *	P → T	67

Notes: Relation between observed posture and spontaneous touch during session 1 and 10. Overall correlation = Pearson correlation coefficient (*r*) of the two waveforms (data points of posture and spontaneous touch (time series of one second) graphed in time order, smoothed with regression (LOESS) function), % Positive windowed correlation = percentage of positive windowed correlations between posture and spontaneous touch in function of time (windows of 30 s), Cross correlation = Maximal correlation coefficient (*r*) of the cross-correlation forecasting analysis [49] with a shifted time window of 30 s relative to each other, P = posture, T = spontaneous touch. Only correlations between −120 and +120 s were considered as meaningful. * moderate correlation (*r* ≥ 0.5). ** high correlation (*r* ≥ 0.7).
